# Assessing the Transferability of Physical Activity Type Detection Models: Influence of Age Group Is Underappreciated

**DOI:** 10.3389/fphys.2021.738939

**Published:** 2021-10-22

**Authors:** Hoda Allahbakhshi, Christina Röcke, Robert Weibel

**Affiliations:** ^1^Department of Geography, University of Zurich (UZH), Zurich, Switzerland; ^2^Digital Society Initiative, UZH, Zurich, Switzerland; ^3^University Research Priority Program (URPP) “Dynamics of Healthy Aging”, University of Zurich, Zurich, Switzerland; ^4^Center for Gerontology, UZH, Zurich, Switzerland

**Keywords:** older adults, physical activity types, real-life, transferability, machine learning

## Abstract

Increasing the amount of physical activity (PA) in older adults that have shifted to a sedentary lifestyle is a determining factor in decreasing health and social costs. It is, therefore, imperative to develop objective methods that accurately detect daily PA types and provide detailed PA guidance for healthy aging. Most of the existing techniques have been applied in the younger generation or validated in the laboratory. To what extent, these methods are transferable to real-life and older adults are a question that this paper aims to answer. Sixty-three participants, including 33 younger and 30 older healthy adults, participated in our study. Each participant wore five devices mounted on the left and right hips, right knee, chest, and left pocket and collected accelerometer and GPS data in both semi-structured and real-life environments. Using this dataset, we developed machine-learning models to detect PA types walking, non-level walking, jogging/running, sitting, standing, and lying. Besides, we examined the accuracy of the models within-and between-age groups applying different scenarios and validation approaches. The within-age models showed convincing classification results. The findings indicate that due to age-related behavioral differences, there are more confusion errors between walking, non-level walking, and running in older adults’ results. Using semi-structured training data, the younger adults’ models outperformed older adults’ models. However, using real-life training data alone or in combination with semi-structured data generated better results for older adults who had high real-life data quality. Assessing the transferability of the models to older adults showed that the models trained with younger adults’ data were only weakly transferable. However, training the models with a combined dataset of both age groups led to reliable transferability of results to the data of the older subgroup. We show that age-related behavioral differences can alter the PA classification performance. We demonstrate that PA type detection models that rely on combined datasets of young and older adults are strongly transferable to real-life and older adults’ data. Our results yield significant time and cost savings for future PA studies by reducing the overall volume of training data required.

## Introduction

According to the World Health Organization, the aging population is increasing rapidly throughout the world (World Health Organization, 2017). This growth in age leads to increasing demands for healthcare services and, consequently, more burden on societies, particularly when older people are trapped in a physically inactive lifestyle (King and King, 2010). Spending too much time on sedentary behavior, such as sitting and lying, has been linked with an increased risk of various physical and mental health disorders, such as chronic diseases, obesity, diabetes, and depression (Bélair et al., 2018; Panahi and Tremblay, 2018; Lavie et al., 2019). Physical activity (PA) is one of the main determinant factors for healthy aging (Voss et al., 2016). PA contributes to maintaining the functional ability and independence of older adults and preventing or mitigating the challenges related to their health status (Gokalp and Clarke, 2013; Taylor, 2014).

An extensive body of literature focused on traditional methods to study the PA behavior of older adults by using questionnaires (Resnick et al., 2008; Verweij et al., 2010; Choi et al., 2017; Martín-María et al., 2020; Takahashi et al., 2020). However, these self-reporting methods are prone to individuals’ recall bias or under/overestimating the duration of activities that have been undertaken and therefore are unable to assess PA behavior reliably as it is actually occurring during human’s daily life (Celis-Morales et al., 2012; Schaller et al., 2016). With the advent of wearable mobile sensor technology, sensor-based methods have been shown to be a powerful tool in addressing the limitations of self-assessed methods by providing valid and reproducible measurements of PA (Liu et al., 2017). However, although sensor-based studies provide essential contributions to the PA field, several shortcomings can still be identified, according to the systematic literature search of our recent review (Allahbakhshi et al., 2019).

First, from a general perspective, existing studies on objectively measured PA primarily focused on PA *level/intensity* (Krüger et al., 2017; McCarthy et al., 2017; Ramires et al., 2017). At the same time, evidence shows that to increase the amount of PA in older adults, providing specific and detailed information about PA has a more significant impact on actual behavior change compared to providing more general PA recommendations (Taylor, 2014). In other words, among the four main dimensions of PA, including frequency, intensity, time, and type, focusing on the *type* of PA and detecting activities, such as sitting, walking, and so on, is of more relevance than mere intensity detection in older adults (Lindemann et al., 2014). Another critical factor is that most of the existing studies that developed PA type detection (PATD) models used data collected under controlled conditions, such as laboratory settings (Bonomi et al., 2009; Freedson et al., 2011; Van Hees et al., 2013; Liu et al., 2017). However, to examine the association between older adults’ PA and their health status, it is vitally important to study their PA behavior in daily life, i.e., in an ecologically valid context (Lindemann et al., 2014). Furthermore, most of the classification models so far have been built based on data collected from limited samples (often less than 30) of healthy younger adults, who might have a different PA performance compared to older adults (Lindemann et al., 2014; Schrack et al., 2016; Allahbakhshi et al., 2019); therefore, the transferability of classification models learned on younger samples to older populations also requires further investigation.

From a methodological perspective, supervised machine learning (ML) algorithms are the most common methods in sensor-based PATD (Adaskevicius, 2014; Bayat et al., 2014; Brondeel et al., 2015; Spinsante et al., 2016; Allahbakhshi et al., 2019). The idea behind these algorithms is learning from training data in which the PA type of each observation is known and then applying this learned model to detect the PA types of a new unknown dataset. However, there are two main challenges for supervised ML algorithms. First, ML assumes the data distribution of the training dataset will never change, whereas this rarely happens in reality due to different physical characteristics and activity performance of the individuals involved in the data collection. Thus, developing a robust ML activity recognition model requires a significant amount of accurately labeled training data. Another challenge, therefore, lies in collecting accurate PA ground truth data in an unobtrusive way, which is time-consuming and challenging, especially for the aging population, as it requires a massive amounts of human workload for data annotation (Diethe et al., 2016; Barbosa, 2018). Thus, providing PATD models that can successfully be transferred to new, unseen datasets—in particular unlabeled data collected on older adults—is essential as it helps reducing the cost and time for data labeling and classification model development and training.

The current study aims to address the identified shortcomings and builds upon our previous work, where we studied real-life PATD in younger adults using accelerometer and global positioning system (GPS) data (Allahbakhshi et al., 2020). In this paper, we aim to investigate to what extent our activity recognition ML algorithm developed on the younger sample can be transferred for predicting the PA types in a sample of older adults. To this end, we collected a new dataset of healthy older adults and conducted extensive analyses both within and between the two age groups by considering different scenarios (e.g., semi-structured vs. real-life). Through our experimental validation, we highlight the limitations of existing methods in assessing the PA behavior of older adults, and we show how the transferability of PATD models to older age groups can be improved.

## Materials and Methods

### Procedure

The experimental study consists of two stages, each concerned with one age group. Stage 1 involved collecting labeled PA type data by 33 healthy young participants (Table 1) who performed seven daily PA types, including lying, sitting, standing, walking on level ground, non-level walking, jogging/running, and cycling, in two different outdoor environments (semi-structured and real-life). The detailed protocol for the data collection has been reported elsewhere (Allahbakhshi et al., 2020). In Allahbakhshi et al. (2020), the PATD was also evaluated on younger adults by developing and testing random forest ML classification models using accelerometer and GPS data from an individual sensor position (individual model) or alternatively integrating sensor data from five different sensor positions (general model) in different scenarios. The developed classification models achieved a high classification performance and transferability on real-life data. Stage 2 of the current study involved collecting PA labeled data from 30 healthy, community-dwelling older adults using a procedure equivalent to Stage 1, aiming to evaluate and improve the transferability of the developed classification models trained on data of younger adults in Allahbakhshi et al. (2020), on new data collected from older adults.

**Table 1:** Physical characteristics of the participants involved in the study.

Physical characteristics	Mean (SD) Younger adults	Mean (SD) Older adults
No. (F/M)	33 (13/20)	30 (17/13)
Age (year)	29 ± (5.6)	72 ± (4.8)
Height (cm)	173 ± (10.05)	167 ± (10.12)
Weight (kg)	67 ± (9.8)	70 ± (15.8)
BMI (kg.*m*^-2^)	22 ± (1.9)	24.5 ± (3.8)

In a procedure equivalent to Allahbakhshi et al. (2020), we collected labeled data from older adults in two protocols, semi-structured and real-life. The older participants conducted the same semi-structured protocol as the younger adults did in Allahbakhshi et al. (2020) in an outdoor environment. However, we eliminated the cycling activity from the list of requested activities for older adults in both protocols due to safety reasons. To simplify the data collection process and avoid putting too much burden on the older participants, we adjusted the real-life protocol. Contrary to younger participants that performed the real-life protocol in an outdoor environment of choice as part of their daily life, we asked older adults to perform the activity protocol in a pre-specified area where they could perform all the requested PA types. However, they were free to perform the activities in their own way and at their own speed, just like to younger participants. During the real-life data collection, an observer followed the older adults and labeled their data, whereas younger participants self-annotated their real-life data using a smartphone app. We asked the older adults to perform the walking activities in a leisure area on two different types of surfaces (paved vs. gravel), including 5min of walking on pavement and a 2min walk on a gravel surface to include as much variation as probably existing in the younger adults’ leisure walking data.

### Device

**Figure 1:**
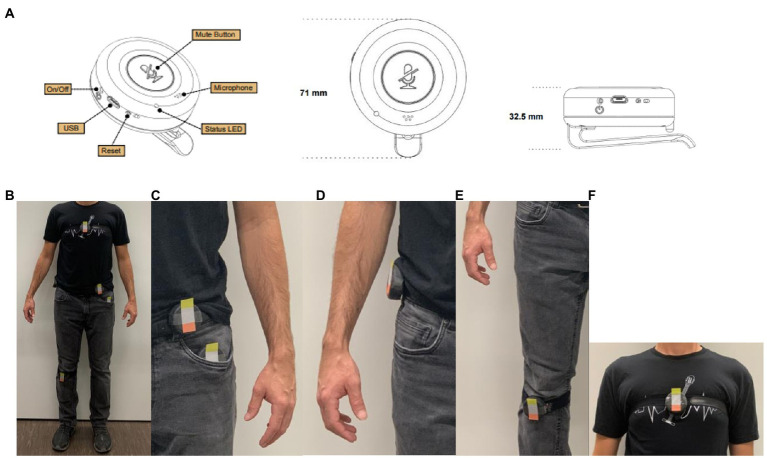
The uTrail device specification and placement, **(A)** the uTrail device, **(B)** the location of uTrail devices on participant’s body, **(C)** left hip and left pocket, **(D)** right hip, **(E)** right knee, and **(F)** chest.

We used the uTrail (firmware versions 6.49 and 6.50) tracking device for the data collection on older adults (Figure 1A), the same device that was also used in Allahbakhshi et al. (2020) for younger adults. The uTrail is a small wearable custom-built device that includes an audio sensor, a GPS sensor (uBlox UC530M) that was set to a sampling rate of 1Hz, and an accelerometer set to a sampling rate of 50Hz that contains three magnetic field channels and three acceleration channels (ST Microelectronics LSM303D; Allahbakhshi et al., 2018).

### Device Placement

The selection of device wearing locations on the body was motivated based on existing studies focusing on a reliable detection of major daily PA types using accelerometer data. For example, the most popular accelerometer placement is on the waist or hip because it is near the body trunk and can thus better represent human movement (Liao et al., 2015). Moreover, findings show that wearing the device on the thigh and chest can help discriminate between sedentary PA types, such as sitting and standing (Skotte et al., 2014), and sitting/standing versus lying (El Achkar et al., 2016), respectively. Barshan and Yüksek (2014) showed that the knee or leg position could provide the best results for detecting daily PA types compared to other parts of the body, such as arms and waist (Barshan and Yüksek, 2014). Therefore, we selected hips, knee, chest, and pocket (representing the thigh position) as the body locations for the device placement.

In total, we mounted five uTrail devices on different body locations (Figure 1B), including left hip inside left pocket (Figure 1C), right hip (Figure 1D), right knee (Figure 1E), and chest (Figure 1F). Two elastic straps, each holding the uTrail, were adjusted around their chest and below their right knee. For the hip positions, we fixed the uTrail devices to their waistband using the device clip.

We applied a direct observation approach for activity annotation using the “aTimeLogger” free app installed on a smartphone for both semi-structured and real-life protocols.

### Participants

A total of 30 healthy older adults aged above 65 participated in the data collection (Table 1). They represent a subset of the Mobility, Activity, and Social Interactions Study (MOASIS), an ambulatory assessment study involving 30days of sensing using the uTrail device in a sample of *N*=153 adults aged 65+ (Röcke et al., 2018). As inclusion criteria, participants were required to be physically and cognitively healthy (MMSE ≥27), walk and run without walking aids (self-report), and accept the instructions of the study protocol. The study was carried out following the rules of the Declaration of Helsinki of 1975 and in compliance with the ethical guidelines of the Faculty of Arts and Social Sciences of the University of Zurich. All participants provided written informed consent.

### Data

The total amount of labeled data collected for older adults was about 115h, consisting of an average of 50h for the semi-structured protocol and 65h for the real-life protocol, respectively (Table 2). In order to have matching PA types between the two age groups, we discarded the cycling activity data from the younger adults’ dataset and updated the amount of their data collected in the previous study (Allahbakhshi et al., 2020) accordingly. The detailed information regarding the distribution of each activity class per age group and per protocol is available in the Supplementary Appendix 1.

**Table 2:** Labeled data collected for the study by the uTrail device.

Dataset	Total Acc. data		Total GPS data		Acc. data per person		GPS data per person	
	Youngeradults	Older adults	Younger adults	Older adults	Younger adults	Older adults	Younger adults	Older adults
Semi-structured	~ 56.2h (10124736)	~ 49.2h (8871854)	~ 54.2h (195160)	~ 51.71h (186184)	~ 1.7h (306810.2)	~ 1.64h (295728)	~ 1.64h (5913.9)	~ 1.72h (6206)
Real-life	~ 66.9h (12045815)	~ 65.3h (11761730)	~ 65.4h (235488)	~ 64.63h (232683)	~ 2.02h (365024.7)	~ 2.17h (392057.7)	~ 1.98h (7,136)	~ 2.15h (7756.1)
Total	~123.1h (22170551)	~ 114.63h (20633584)	~119.6h (430,648)	~116.35h (418867)	~ 3.73h (671834.9)	~ 3.82h (687785.7)	~ 3.62h (13049.9)	~ 3.87h (13962.1)

*Numbers in brackets denote the number of sample points.*

### Data Preprocessing

We preprocessed the accelerometer and GPS data using the approach presented in (Allahbakhshi et al., 2020). We then derived, as explained in (Allahbakhshi et al., 2020), a set of 85 time and frequency domain features from the accelerometer data, as well as two GPS features (average speed and elevation difference) from the GPS data, all within overlapping 2s time windows. A flowchart of the accelerometer and GPS signal processing steps is available in the Supplementary Appendix 2.

The time and frequency domain features from accelerometer data are the same and extended features, respectively, as those introduced by Kwapisz et al. (2011) and Bao and Intille (2004), which have already been shown to be informative for PATD models using accelerometer data. They include as:

1. Time domain features: mean, standard deviation, and range of three axes and total acceleration, correlation among three axes, kurtosis, skewness, and average absolute difference of three axes, number of observations falling within each of 10 bins of the three axes, and time interval between local peaks and number of peaks of three axes.
2. Frequency domain features using FFT: power spectral density, energy of the signal, mean of the first three dominant frequencies, and amplitude of the first three dominant frequencies of three axes and total acceleration.

We aggregated the labeled PA types into six classes, including lying, sitting, standing, walking, non-level walking, and running. We used ArcGIS v.10.6.1 and the R statistical computing software for the data analysis (R Core Team, 2013).

### Classification Model Development

As in the previous study (Allahbakhshi et al., 2020), we used a random forest classifier for the PA type classification. Random forests are a representative of so-called ensemble classifiers, which build a classification model by aggregating the predictions of multiple individual classifiers and thus tend to be more flexible and robust compared to individual models. More specifically, RF is a bagging ensemble classifier that builds multiple individual decision trees in a parallel way; each model is built based on a random subset of the training feature data (Zhang and Ma, 2012).

We created three different training datasets, one using data from the semi-structured protocol only, one using data from the real-life protocol only, and one using the combined dataset of both the semi-structured and real-life protocols. We used the RF classifier to build the classification models in different scenarios, which featured a particular combination of training dataset, validation method, and test data (Table 3). For each scenario, we examined both single (accelerometer data only) and multi-sensor (accelerometer and GPS data) approaches to build the RF classification models. We built a general model that was trained with data obtained from all five sensor positions (chest, left hip, right hip, left pocket, and right knee) and also five individual models, each trained with data from a particular single-sensor position.

**Table 3:** Scenarios for separating data into training and test datasets and the corresponding validation method.

Scenario no.	Training dataset	Validation method and test data
Scenario 1	Semi-structured dataset	(A) L1SO cross-validation on semi-structured data (B) L1SO cross-validation on real-life data
Scenario 2	Combined (semi-structured and real-life) dataset	(A) L1SO cross-validation on combined data (B) L1SO cross-validation on real-life data
Scenario 3	Real-life dataset	(A) L1SO cross-validation on real-life data

To assess whether classification performance differences exist between the two age groups, we built two within-age models, each using data from one age group (Table 4). To assess the transferability of the PA classification models trained with younger adults’ data on data from the older group, we built the Young-trained-between-age (Y-trained-btw-age) models. To assess how the transferability of the classification models on the data of older adults can be improved, we created the Young and Old-trained-between-age (YandO-trained-btw-age) model, which was trained with a combined dataset of both age groups and tested on the older adults’ data.

**Table 4:** The developed models to evaluate the classification performance differences within and between the two age groups.

Model	Training dataset	Testing dataset
Within-old-age	Older adults	Older adults
Within-young-age	Younger adults	Younger adults
Y-trained-btw-age	Younger adults	Older adults
YandO-trained-btw-age	Younger and older adults	Older adults

### Evaluating the Effects of the Validation Strategy and the Classifiers Used

In order to evaluate the effects of choices concerning the methods used for PA type classification, we further evaluated the effect of the choice of cross-validation strategy, and the effect of the classifier algorithm used on the classification results that can be obtained. The corresponding results are reported in the Supplementary Appendix 3. The codes developed during the current study are available from the GitHub repository: https://github.com/Hoda-Bakhshi/PA-tracking-sensor-data.

## Results

We present the overall accuracies of the within-and between-age RF models—both for the general model (using data from all sensor positions) and the individual, single-sensor models—as evaluated using both the Leave-one-subject-out (L1SO) cross-validation strategy and validation with the real-life dataset, for the three scenarios given in Table 3. For the sake of brevity and conciseness, we show the classification results of only the general models in Sections Results for Within-Age Models and Results for Between-Age Models. The figures showing the classification results of the individual models are available in the Supplementary Appendix 4.

### Results for Within-Age Models

**Figure 2:**
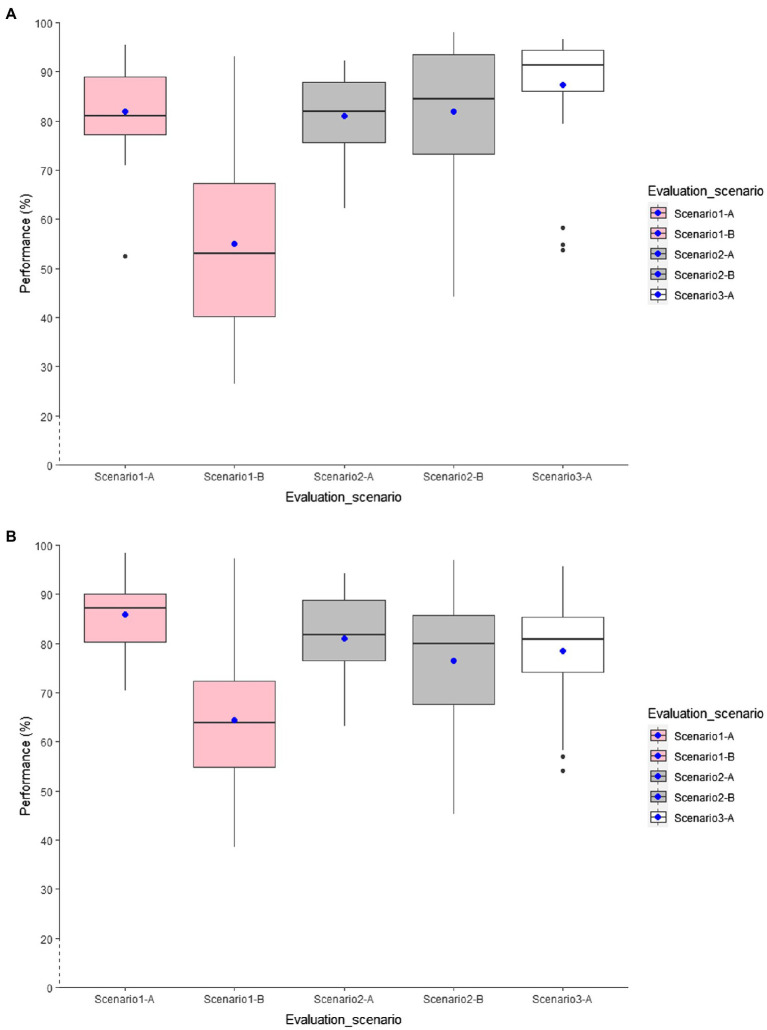
Distribution of overall accuracy for the general accelerometer-based RF classification models. **(A)** within-old-age models and **(B)** within-young-age models.

In Scenario 1-A, using L1SO cross-validation (with training data) and accelerometer data only, all models generated with older adults’ data except the chest model performed worse than the models trained with younger adults’ data (Figure 2). For example, the general model of older adults achieved 82% accuracy, whereas the general model of younger adults came to 86% accuracy. In both within-age models, the general models outperformed the individual models. Among the individual models, the knee position scored highest with 77 and 82% accuracy in the within-age model for older and younger adults, respectively. Both within-age models trained with semi-structured data showed a significant weak classification transferability on the real-life dataset. However, the within-young-age models achieved higher overall accuracy than the result obtained by the within-old-age model when tested on the real-life data.

**Figure 3:**
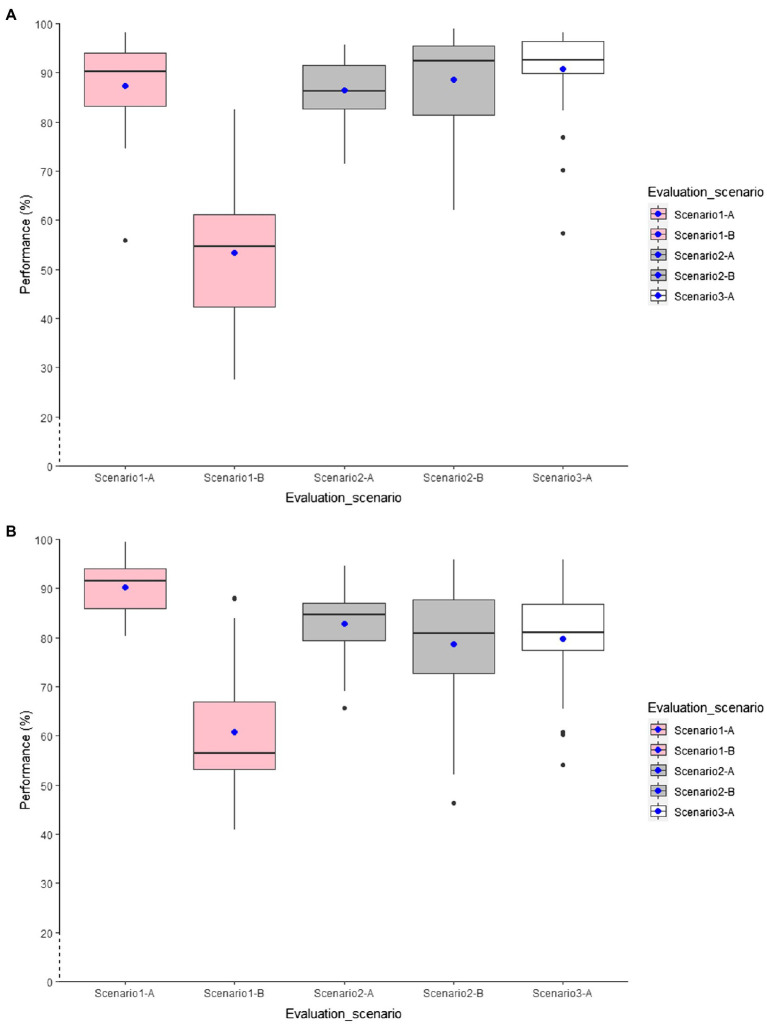
Distribution of overall accuracy for the general accelerometer and GPS-based RF classification models. **(A)** within-old-age models and **(B)** within-young-age models.

Adding GPS features to the accelerometer features improved the classification performance for both within-age models validated by L1SO by up to 7% (Figure 3). However, similar to the accelerometer-based models, the classification performance decreased for all within-age models when tested on the real-life data (Figure 3).

As Scenario 1 used data collected using the semi-structured protocol, which was the same protocol for both age groups, we compared the classification performance metrics of a participant who obtained the highest GPS contribution in Scenario 1-A, using L1SO cross-validation (with training data) for both age groups (Tables 5, 6). The classification metrics in both within-age models show that the activities *non-level walking* and *walking* achieved the lowest rates when using accelerometer data only and L1SO validation (with training data), and adding GPS features considerably improved the classification performance for these activities, particularly for the older participant (Tables 5, 6).

**Table 5:** Classification metrics of an older participant (with the highest GPS contribution) in Scenario 1-A.

Activity types	ACC-based model			ACC+GPS-based model		
	Precision	Recall	F1	Precision	Recall	F1
Lying	100	100	100	100	100	100
Non-level Walking	64.73	100	78.59	84.63	98.53	91.06
Running	90.70	99.15	94.74	94.40	100	97.12
Sitting	100	100	100	100	100	100
Standing	100	100	100	100	100	100
Walking	100	20.72	34.32	97.34	72.91	83.37

**Table 6:** Classification metrics of the participant from the younger age group (with the highest GPS contribution) in Scenario 1-A.

Activity types	ACC-based model			ACC+GPS-based model		
	Precision	Recall	F1	Precision	Recall	F1
Lying	100	99.13	99.56	100	99.12	99.56
Non-level Walking	88.01	59.24	70.81	96.92	95.45	96.18
Running	99.12	99.12	99.12	100	98.25	99.12
Sitting	99.12	100	99.56	99.12	100	99.56
Standing	100	100	100	100	100	100
Walking	51.76	84.43	64.17	91.24	94.63	92.90

In Scenario 2, we trained the classification models based on the combined dataset of semi-structured and real-life protocols. Similar to Scenario 1-A, using L1SO cross-validation (with training data) and accelerometer features only, individual models trained with younger adults’ data achieved a higher accuracy, except for the right-hip model that obtained the same results in both within-age models. Both age groups achieved the same classification performance of 81% accuracy for their general models. Contrary to Scenario 1-B, where the performance dramatically dropped with real-life data, this scenario (Scenario 2-B) showed a considerable classification transferability on the real-life dataset for all models.

Adding GPS features to the accelerometer features improved the classification performance for all models validated by L1SO of the training dataset (Scenario 2-A) up to 8 and 3% in the within-age model for older and younger adults, respectively. This also resulted in stable classification performance with real-life data (Scenario 2-B). Moreover, adding GPS features increased the classification performance by up to 4% for the older adults’ models using L1SO with real-life data (Scenario 2-B) compared to with training data (Scenario 2-A), whereas conversely it reduced the classification accuracy up to 5% for the younger adults’ models.

In Scenario 3, where we used only the real-life data for training the classifier (Scenario 3-A), all models trained with the data of older adults outperformed those trained with the data of the younger adults. Furthermore, GPS features contributed to improving the within-old-age models by up to 8%, while this number was limited to 3% for within-young-age models.

### Results for Between-Age Models

This section reports to what degree models trained on data of younger adults can be transferred to the data of older adults (Table 4). In the Y-trained-btw-age model, we used the younger adults’ data for training and the older adults’ data for testing the classification model. In the YandO-trained-btw-age model, we treated all data collected by older and younger participants as a single dataset, trained the classification models based on that, and then tested the developed models on unseen data of older adults.

**Figure 4:**
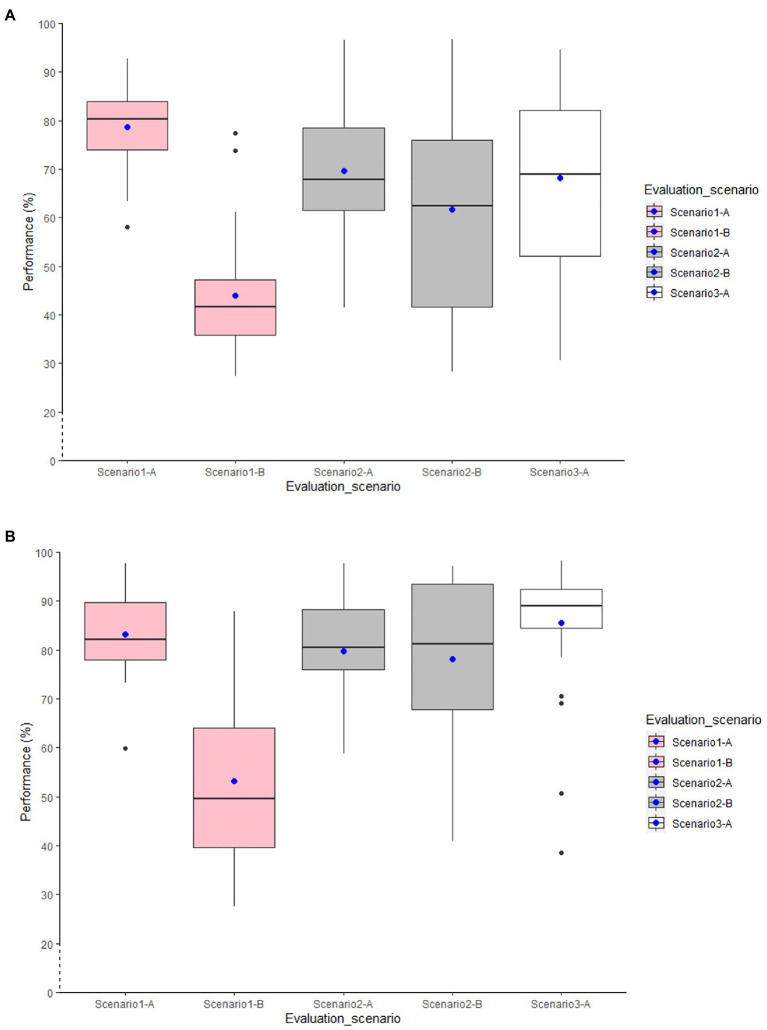
Distribution of overall accuracy for the general accelerometer-based RF classification models. **(A)** Y-trained-btw-age models and **(B)** YandO-trained-btw-age models.

Using L1SO cross-validation (with training data) and accelerometer data only in Scenario 1-A, all YandO-trained-btw-age models using the combined training data performed better than the Y-trained-btw-age models, which used young for training and older for testing (Figure 4). Adding older adults’ data to the younger adults’ data increased the classification performance by 4% for the general model and by up to 8% for the individual YandO-trained-btw-age models. The knee models outperformed the other individual models with 76 and 81% overall accuracy for the Y-trained-btw-age and YandO-trained-btw-age models. Similar to the within-age models in Scenario 1-B, the between-age models showed a high classification transition error on real-life data. Moreover, some individual models scored higher in accuracy than the general models when tested on real-life data.

**Figure 5:**
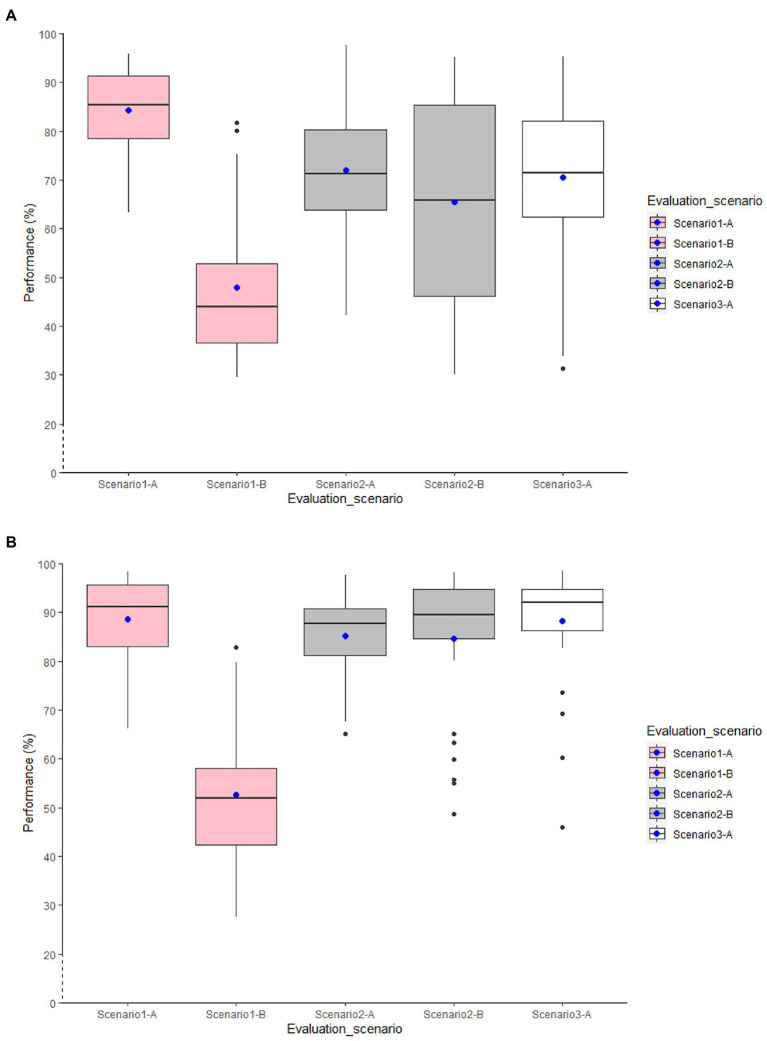
Distribution of overall accuracy for the general accelerometer and GPS-based RF classification models. **(A)** Y-trained-btw-age models and **(B)** YandO-trained-btw-age models.

Adding GPS features (Figure 5) contributed to improving the classification performance by up to 6% for individual models and by 5 and 6%, respectively, for the general Y-trained-btw-age and YandO-trained-btw-age models validated by L1SO cross-validation of the training dataset (Figure 5; Scenario 1-A). However, the ACC+GPS models also did not show convincing results with real-life data (Figure 5; Scenario 1-B).

In Scenario 2, using accelerometer data only (Figure 4), all models trained with combined data of both age groups scored higher in accuracy than the models trained with younger adult’s data only. Using L1SO cross-validation (with training data) and accelerometer data only (Scenario 2-A), as in Scenario 1-A, the knee models were the best among the individual models. The general YandO-trained-btw-age model with 80% accuracy obtained 10% more transferability on older adults’ data than the general Y-trained-btw-age model. This transferability difference between two general models increased to 16% with real-life data (Figure 4; Scenario 2-B).

ACC+GPS models (Figure 5) improved the classification performance for all models, using L1SO cross-validation with training (Scenario 2-A) and real-life data (Scenario 2-B). However, adding GPS features to the accelerometer features resulted in more stable classification performance for YandO-trained-btw-age models than for the Y-trained-btw-age models when tested on real-life data (Scenario 2-B).

Similar to the first two scenarios, the between-age models with age-combined data performed better than the between-age models with age-opposing data in Scenario 3, when trained and tested with real-life data only. Using multi-sensor data increased the classification performance by up to 7 and 4% in the YandO-trained-btw-age and the Y-trained-btw-age models, respectively.

## Discussion

The main aim of this study was to investigate the transferability of PATD models trained with younger adults’ data in detecting major daily living activity types of older adults representing postures (lying, sitting, and standing) and motion-related PA types (level walking, non-level walking, and running/jogging). Furthermore, we explored various scenarios to evaluate the classification performance within and across the two age groups.

### Within-Age Group PA Type Detection

#### Scenario 1

We built two within-age models in three different scenarios to compare the behavioral differences in two distinct age groups. The results show that when the same study setting is used for data collection for both groups, the younger adults’ models outperform the older adults’ models. Comparing the performance of the general models of the two participants who achieved the highest GPS contribution in Scenario 1-A in their respective age group, we found no confusion between motion and posture activities for both participants. Both general models trained with or without GPS data detected the activities lying, sitting, and standing with a high f-score of above 99%, showing no posture classification difference between older and younger participants. However, significant misclassification existed between the activities level walking and non-level walking, especially in accelerometer-only models.

Moreover, we noted that there was confusion between running and level/non-level walking activities for older adults who achieved low overall classification accuracy, which did not occur for the younger group. This is in line with the observation of Wang et al. (2015) that there are behavioral differences in motion activity performance between older and younger adults, respectively. Moreover, it supports the results by Wang et al. (2015) that older adults run slower than younger adults, which might be due to the consequences of aging, such as the reduction of muscle strength, agility, and endurance. Therefore, their running activity performance may generate similar movement data profiles to their walking behavior and mislead the classifiers. Adding GPS features showed a consistent contribution in improving both the performance of within-age models, particularly by reducing the confusion errors between level and non-level walking activities. However, adding GPS features to the accelerometer features produced more generalization errors for younger adults when tested with the real-life dataset (Scenario 1-B) because they performed the real-life protocol in different urban and leisure surroundings.

#### Scenario 2

Using the combined, accelerometer-only dataset for training the within-age models and L1SO cross-validation with training data (Scenario 2-A), the general accelerometer-only models of both age groups achieved the same overall accuracy. Adding GPS features, the older adults’ general model obtained 3% more accuracy than the younger adults’ model. Besides, using L1SO cross-validation with real-life data (Scenario 2-B), the older adults’ general model outperformed the younger adults’ model and was strongly transferable to the real-life dataset, mainly when adding GPS features to the accelerometer features. Compared to the older adults’ real-life dataset, there were more variations in the dataset for younger adults, explaining the overall decrease in accuracy in their classification models, especially when validated by L1SO cross-validation with real-life data (Scenario 2-B). The real-life environment used by older adults was limited to a particular area that was the same for all participants, while the younger participants were allowed to choose their own real-life environment to perform their activities. This contributed to increased variation in environmental factors, such as surface types used for level walking or steepness of slopes for non-level (uphill, downhill) walking, ultimately causing lower classification performance.

Compared to our previous study (Allahbakhshi et al., 2020), we detected fewer activity types and achieved slightly lower classification performance for the younger participants. This might be due to discarding the cycling activity, which resulted in less training data. Moreover, the cycling activity was the most distinguishable activity type as it produces distinctive movement profiles in the accelerometer data and recognizable cyclic patterns, especially in the knee sensor data (Allahbakhshi et al., 2020).

#### Scenario 3

As another extension to our previous study, we added Scenario 3 to show that using only real-life data could be sufficient to generate classification models with convincing results for predicting PA types collected in the real-life environment of an unseen participant. The discussion of Scenario 2 mentioned the difference in real-life protocols for the two age groups, which explains why the classification models for older adults outperformed those for younger adults also in Scenario 3. Similar to Scenario 2, adding GPS features provided a higher contribution in improving the results for older adults since there was a lower number of missing values and noisy data in their real-life GPS data compared to younger participants. This supports one of the conclusions in our previous paper that the high performance of the developed PATD models can only be achieved when high-quality GPS and map-matching data are available (Allahbakhshi et al., 2020).

### Between-Age Group PA Type Detection

To examine the extent to which our classification models can predict older adults’ daily PA types, we built and tested two between-age models. In general, the Y-trained-btw-age models trained with data of younger adults only were weakly transferable to the older adults’ data. Comparing the Y-trained-btw-age models with within-old-age models, the developed Y-trained-btw-age models showed a dramatic decrease in Scenarios 2 and 3, mainly when evaluated with L1SO cross-validation applied to the real-life dataset. However, they achieved lower transition errors and higher transferability on older adults’ data in Scenario 1. Therefore, we conclude that the transition errors arise both from the age-behavioral differences between the two age groups and the variations in the real-life study settings.

Based on the results of Ermes et al. (2008) and our previous paper (Allahbakhshi et al., 2020), we realized that in order to improve the transferability of classification models for a real-life dataset, labeled real-life data should be included in the training data (Scenario 2). We applied the same logic to increase the transferability of our classification models to the data of older adults. We developed the YandO-trained-btw-age models by creating an integrated dataset of both younger and older adults. The YandO-trained-btw-age models showed the most consistent accuracy with within-old-age models when evaluated by L1SO cross-validation of both training and real-life data in Scenario 1, followed by Scenarios 3 and 2, respectively. We, therefore, conclude that the new models trained with combined datasets of both age groups generate robust models with reproducible classification performance when applied to data of older adults.

### Individual Classification Models

The individual models underperformed the general models, where data from all sensor positions are used. The possible reasons for the lower performance of individual models could be the inability of a single-sensor position in providing sufficient information for detecting all daily PAs and the effects of signal noise and motion artifacts due to the non-rigid attachment of the sensors to the body, masking the intended signal. We applied different measures to reduce or eliminate these effects: For example, we asked participants to use tight clothing, such as pants with tight pockets (for the pocket/thigh position), placed sensors in locations affected minimally by body motion (hip positions) and strapped the devices firmly to their body (chest and knee positions). However, some cases possibly led to flipping or rotating the device during activity performance, affecting the data quality, and classification performance. For instance, some participants wore pants with big and loose pockets. Therefore, the material, tightness, size, shape, and orientation of the pockets would vary. This also explains why the pocket model performed worst compared to the other individual models. Besides, despite having full control over device placement during the semi-structured data collection, it happened that after some activities, such as running, the knee, and chest-mounted devices, were slightly displaced or flipped due to the rapid leg or upper body movement. This might have caused further issues during real-life activity performance, where participants’ poor control of the device placement could result in unpredictable extraneous motion data and data quality issues.

Further, there was no instruction regarding how to perform the PAs during real-life data collection. Therefore, participants might have performed posture activities differently, influencing accelerometer data from different body locations (Hughes et al., 2020). For example, staying in a sitting position with legs crossed rather than legs straight can change the accelerometer values for the knee-positioned sensor. Participants might have performed minor twitches during standing or sitting activities or laid in a lateral body position rather than staying in a prone, or supine position, affecting accelerometer signals to be different in different body locations.

Among the individual classification models, the knee-positioned models were the best in most of the validation scenarios and could achieve accuracy levels comparable to those of their corresponding general models. Even though there were a few cases where the chest or hip models achieved the same or slightly higher accuracy than the knee model, the number of occurrences in which the knee model outperformed other individual models was higher. This is possibly because the knee position can better measure the periodic leg motion and capture the signal characteristics representing the cyclic nature of certain types of motion activities, such as walking and running. Therefore, we conclude that the knee model provides a minimal, non-intrusive device configuration with reliable activity type recognition accuracy for both younger and older adults.

### Limitations and Future Work

This study has some limitations that should be addressed in future research. A limitation of this work is the selection bias of the MOASIS study (Röcke et al., 2018) that served as our sample, which focused on older adults with good physical and cognitive health. Involving older adults with various functional levels, including those requiring walking aids, should be considered in future research to capture more fully the heterogeneity of PA types and patterns in the older population. Though we investigated the effects of various factors on activity recognition models, assessing the influence of environmental factors, such as weather, is missing and requires a separate study. We obtained a high classification performance for detecting daily PA types using the RF classifier. However, applying advanced technologies for preprocessing raw accelerometer data, such as the reduction of the integration drift (Zhao, 2018), might help to further improve the classification performance. Further, since we applied the random forest method as a classifier, which performs feature selection throughout the classification process, we did not apply any feature engineering method. However, as shown in Fujiki et al. (2009), there might be collinearity in accelerometer data from different body locations. Therefore, applying advanced classification models and feature engineering methods might help gain further insights into the most informative features derived from each sensor location and contribute to further improving the results of the present work. Finally, adjusting the classification models to perform in real-time, which would be important in the context of health-monitoring systems, is another challenge that should be addressed in future studies.

## Conclusion

In this study, we assessed the influence of age in the performance and transferability of PATD models, which has so far been understudied in the existing literature. Our results led to the following findings:

1. The performance of PATD models is satisfactory if staying within-age groups and study settings.
2. While most studies on PATD used only samples of younger adults who performed PAs in controlled conditions, we showed that the transferability of classification models using such datasets is actually weak, particularly when applied in older adults and real-life settings.
3. The transferability of PATD models to real-life data considerably improves by creating a training dataset with combined data of semi-structured and real-life settings.
4. Creating a training dataset with a mixture of younger and older participants improves the transferability of PATD between-age models on older adults’ data significantly and brings it to the level seen in within-old-age group classification models.
5. The ACC+GPS knee model provides the best single-device configuration for both age groups, supporting a non-intrusive model for long-term real-life PA monitoring, particularly for older adults.
6. Overall, we believe that our work has delivered insights that should help others who are designing PATD studies, in particular ones focusing on older adults and real-life settings, reducing the cost and time required for data labeling and classification model development and training.

## Data Availability Statement

The original contributions presented in the study are included in the GitHub repository: https://github.com/Hoda-Bakhshi/PA-tracking-sensor-data and article/Supplementary Material, further inquiries can be directed to the corresponding author.

## Ethics Statement

Ethical review and approval was not required for the study on human participants in accordance with the local legislation and institutional requirements. The patients/participants provided their written informed consent to participate in this study. Written informed consent was obtained from the individual(s) for the publication of any potentially identifiable images or data included in this article.

## Author Contributions

HA and RW: conceptualization and methodology. HA: data curation, formal analysis, investigation, software, validation, visualization, and writing—original draft. HA: resources and writing—review and editing in coordination with CR and RW. RW: supervision. All authors contributed to the article and approved the submitted version.

## Funding

This work was supported in part by the University Research Priority Program, the “Dynamics of Healthy Aging” of the University of Zurich and the Velux Stiftung (grant no. 917) by providing the equipment for data collection and recruiting of older participants, as well as the University of Zurich for funding the open access publication fee.

## Conflict of Interest

The authors declare that the research was conducted in the absence of any commercial or financial relationships that could be construed as a potential conflict of interest.

## Publisher’s Note

All claims expressed in this article are solely those of the authors and do not necessarily represent those of their affiliated organizations, or those of the publisher, the editors and the reviewers. Any product that may be evaluated in this article, or claim that may be made by its manufacturer, is not guaranteed or endorsed by the publisher.

## Abbreviations

GPS: Global positioning system
L1SO: Leave one subject out
ML: Machine learning
PA: Physical activity
PATD: Physical activity type detection
YandO-trained-btw-age: Young and old-trained-between-age
Y-trained-btw-age: Young-trained-between-age
