# Supplementary material for: Assessing the Transferability of Physical Activity Type Detection Models: Influence of Age Group Is Underappreciated
Source: Front Physiol. 2021 Oct 22;12:738939. doi: 10.3389/fphys.2021.738939 (PMC8569407; doi:10.3389/fphys.2021.738939)
Supplement: Supplementary file 1 [file Data_Sheet_1.docx]

Supplementary Material

**Appendix 1.** Statistics of labeled physical activity data

**Table A1.1.** Statistics of labeled data of younger adults

|  | **Semi-structured data (%)** | | **Real-life data (%)** | |
| --- | --- | --- | --- | --- |
| **Activity class** | **Accelerometer** | **GPS** | **Accelerometer** | **GPS** |
| Lying | 10.24 | 10.52 | 5.22 | 5.24 |
| Non level-walking | 33.00 | 35.66 | 25.64 | 26.09 |
| Running | 9.72 | 10.05 | 11.77 | 11.81 |
| Sitting | 10.64 | 10.44 | 5.98 | 5.92 |
| Standing | 10.56 | 9.30 | 5.23 | 5.03 |
| Walking | 25.85 | 24.02 | 46.16 | 45.91 |

**Table A1.2** Statistics of labeled data of older adults

|  | **Semi-structured data (%)** | | **Real-life data (%)** | |
| --- | --- | --- | --- | --- |
| **Activity class** | **Accelerometer** | **GPS** | **Accelerometer** | **GPS** |
| Lying | 10.82 | 10.01 | 3.59 | 3.61 |
| Non level-walking | 32.36 | 34.83 | 19.95 | 20.19 |
| Running | 12.26 | 13.68 | 7.70 | 7.72 |
| Sitting | 9.56 | 9.18 | 3.52 | 3.58 |
| Standing | 10.70 | 10.03 | 3.96 | 4.02 |
| Walking | 24.31 | 22.28 | 61.28 | 60.88 |

**Appendix 2.** Flowchart of accelerometer and GPS signal processing

**Figure A2.1.** Flowchart of accelerometer and GPS signal processing

**Appendix 3.** Evaluating the effects of the validation strategy and the classifiers

# Evaluating the effects of the validation strategy and the classifiers

In order to evaluate the effects of choices concerning the methods used for PA type classification, we further evaluated

1) the effect of the choice of cross-validation strategy, and

2) the effect of the classifier algorithm used on the classification results that can be obtained.

To keep things short, we only report the results regarding a single type of models, that is, the best-performing classification models: the general models applied within age groups.

## Effect of cross-validation strategy

In addition to the L1SO evaluation reported in the main paper, we evaluated the general within-age random forest (RF) classification models using two subject-based k-fold cross-validations. In our first subject-based 10-fold cross-validation, the data of 90% of the subjects were used for training, and the remaining data of 10% of the unseen subjects were used in turn for validation. The second subject-based 5-fold cross-validation used 80% - 20% dataset splitting. That is, we used the data of 80% of the subjects for training and the data of 20% of the unseen subjects for validation. This was repeated ten and five times for subject-based 10-fold and 5-fold cross-validations, respectively, selecting different subjects for testing in each repetition.

We observed that L1SO has the best performance, followed by subject-based 10-fold (90%-10%), with slightly different rates, and 5-fold (80%-20%) (Table A3.1). However, the models trained with the data of younger adults showed less variation of the overall classification accuracy for all validation methods than those trained with older adults’ data. The maximum difference between L1SO and k-fold cross-validation performance is 2 % for within-young-age models (e.g., in ACC+GPS, S1-real-life). In comparison, this range increased to 4% for within-old-age models (e.g., in ACC+GPS, S1-real-life).

**Table A3.1.** Overall classification performance of RF models based on different cross-validation techniques for general within-age models

| **Sensor type(s)** | | **Accelerometer only** | | | | | **ACC+GPS** | | | | |
| --- | --- | --- | --- | --- | --- | --- | --- | --- | --- | --- | --- |
| **Scenario** | | **S1** | | **S2** | | **S3** | **S1** | | **S2** | | **S3** |
|  | **Cross-validation** | **Semi-structured**  **data** | **Real-life data** | **Combined data** | **Real-life data** | **Real-life data** | **Semi-structured**  **data** | **Real-life data** | **Combined data** | **Real-life data** | **Real-life data** |
| **General**  **within-old-age model** | L1SO | 82 | 55 | 81 | 82 | 87 | 86 | 53 | 86 | 89 | 91 |
|  | 10-fold (90%-10%) | 81 | 53 | 82 | 83 | 88 | 87 | 49 | 87 | 90 | 92 |
|  | 5-fold (80%-20%) | 81 | 53 | 78 | 78 | 85 | 86 | 52 | 84 | 86 | 90 |
| **General**  **within-young-age model** | L1SO | 86 | 64 | 81 | 76 | 78 | 90 | 61 | 83 | 79 | 80 |
|  | 10-fold (90%-10%) | 86 | 64 | 80 | 76 | 80 | 90 | 59 | 83 | 79 | 82 |
|  | 5-fold (80%-20%) | 86 | 63 | 81 | 76 | 78 | 88 | 61 | 82 | 78 | 80 |

The generally low variation of the overall classification accuracies indicates that the subject-based k-fold cross-validation leads to realistic results. In our previous paper (Allahbakhshi et al., 2020), we had discarded the use of traditional 10-fold cross-validation, that is, dividing all the data corresponding to the given scenario into ten partitions regardless of the subject or class they belong to. This cross-validation strategy led to artificially high classification performance scores due to model overfitting. However, using the subject-based k-fold cross-validation, we did not allow data from the same participant to appear in both the test and training set and thus prevented the classification models from overfitting. Regarding the processing time, subject-based k-fold cross-validation was faster than L1SO. Therefore, we recommend using subject-based k-fold (typically: 10-fold) cross-validation as a fair compromise for PA type detection applications that focus on investigating inter-individual differences and require high computational performance in model development. However, contrary to L1SO, subject-based k-fold cross-validation is unable to provide detailed information about model performance at the individual level, as multiple participants will occur per partition. For instance, if the number of participants is 30, while the number of partitions is 10 in a subject-based 10-fold cross-validation, each partition will contain three participants, making it impossible to resolve the model performance at the individual level. Hence, if the evaluation of intra-individual model performance is of importance, L1SO cross-validation would still be the preferred strategy.

## Gradient boosting versus Random forest classifiers

The two most popular ensemble methods are bagging and boosting (Zhang and Ma, 2012). Therefore, in addition to using random forests (RF), a bagging ensemble model, we tested another ensemble classifier, gradient boosting (GB), which uses boosting as the method for building ensemble classification models. The boosting ensemble model sequentially trains individual models and learns from mistakes made by the previous model. We applied both ensemble classifiers to the general within-age models. We used the ‘ranger’ and ‘xgboost’ packages in the R statistical computing software for random forest and gradient boosting classifiers, respectively (Chen, T., et al., 2015; Wright and Ziegler, 2017). Both packages showed a significantly faster processing time than other comparable packages, such as ‘randomForest’ or other boosting packages in R.

We compared the performance of the two ensemble classifier algorithms in three scenarios, using L1SO cross-validation with training data and real-life data (Table A3.2). The results show that both RF and GB classifiers achieved almost the same overall accuracy for within-old-age models in each scenario, except for Scenario 1, using L1SO with real-life data, where GB performed > 5% better than RF for both single and multi-sensor models. Similar classification performance was seen for general within-young-age models. However, GB outperformed RF only in Scenario 1, using L1SO with real-life data when trained with the younger adults’ accelerometer data.

Testing the general RF models with GB supports the results by Lee and Kwan (Lee and Kwan, 2018) that both classifiers achieve similarly convincing PA type detection results. There were only few cases where GB outperformed RF. However, GB has more hyperparameters to be tuned compared to RF, thus increasing the usage complexity. Moreover, considering the processing time, GB was slower than RF. In conclusion, RF represents a very valuable choice of classifier for the applications targeted in the type of study reported in this paper.

**Table A3.2**. Overall classification performance using different ensemble classifier alrgorithms for general within-age models

| **Sensor type(s)** | | **Accelerometer only** | | | | | **ACC+GPS** | | | | |
| --- | --- | --- | --- | --- | --- | --- | --- | --- | --- | --- | --- |
| **Scenario** | | **S1** | | **S2** | | **S3** | **S1** | | **S2** | | **S3** |
|  | **Classifier** | **L1SO** | **Real-life** | **L1SO** | **Real-life** | **L1SO** | **L1SO** | **Real-life** | **L1SO** | **Real-life** | **L1SO** |
| **General**  **within-old-age**  **model** | Random forest | 82 | 55 | 81 | 82 | 87 | 87 | 53 | 86 | 89 | 91 |
|  | Gradient boosting | 82 | 60 | 82 | 82 | 88 | 88 | 61 | 88 | 89 | 94 |
| **General**  **within-young-age model** | Random forest | 86 | 65 | 81 | 76 | 78 | 90 | 61 | 83 | 79 | 80 |
|  | Gradient boosting | 86 | 70 | 80 | 76 | 76 | 92 | 61 | 83 | 79 | 78 |

# References

Allahbakhshi, H., Conrow, L., Naimi, B., Weibel, R. (2020). Using Accelerometer And GPS Data for Real-Life Physical Activity Type Detection. Sensors 20:588. https://doi.org/10.3390/s20030588

Chen, T., He, T., Benesty, M., Khotilovich, V., & Tang, Y. (2015). Xgboost: Extreme Gradient Boosting. R Packag. version 0.4-2, 1–4.

Lee, K., Kwan, M.P., (2018). Physical activity classification in free-living conditions using smartphone accelerometer data and exploration of predicted results. Comput. Environ. Urban Syst. 67, 124–131. https://doi.org/10.1016/j.compenvurbsys.2017.09.012

Wright, M.N., Ziegler, A., (2017). Ranger : A Fast Implementation of Random Forests for High Dimensional Data in C ++ and R. J Stat Softw. 77. https://doi.org/10.18637/jss.v077.i01

Zhang, C., Ma, Y. (2012). Ensemble Machine Learning: Methods and Applications. Springer Science & Business Media. https://doi.org/10.1007/978-1-4419-9326-7

**Appendix 4.** Distribution of overall accuracy for the within- and between-age RF individual, single-sensor classification models— as evaluated using both the Leave-one-subject-out (L1SO) cross-validation strategy and validation with the real-life dataset, for the three scenarios given in Table 3 of the main article.


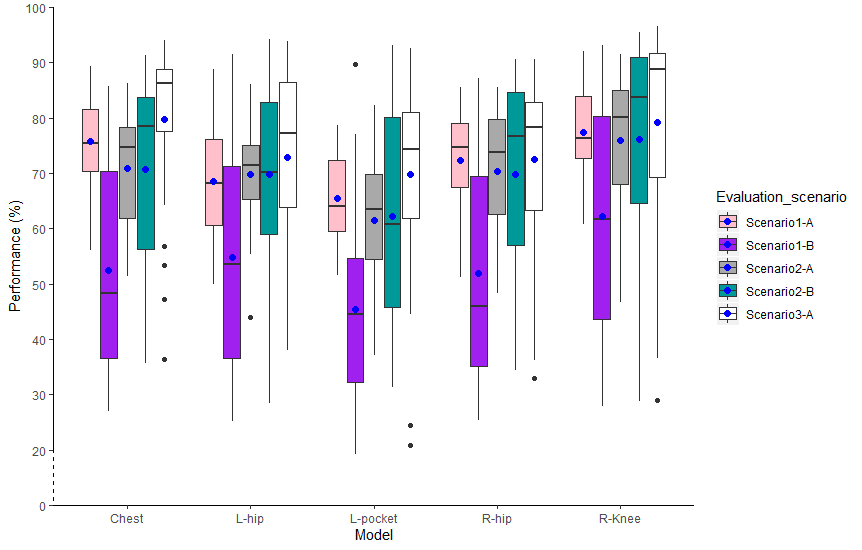


**A**


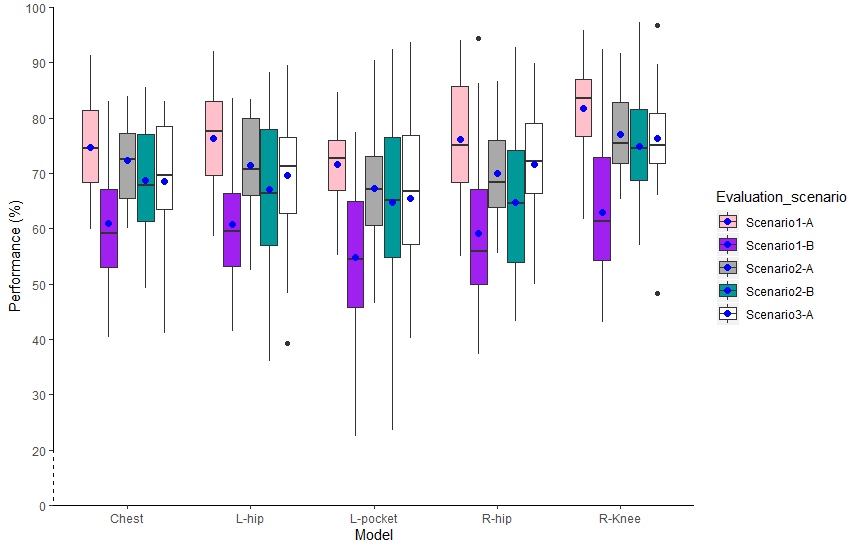


**B**

**Figure A4.1.** Distribution of overall accuracy for the individual accelerometer-based RF classification models. **(A)**: within-old-age models, **(B)**: within-young-age models.

**
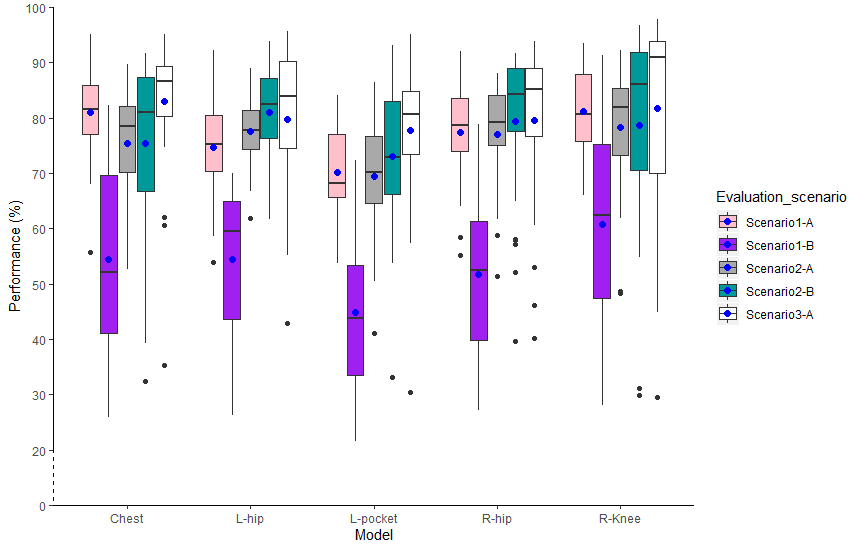
**

**A**

**
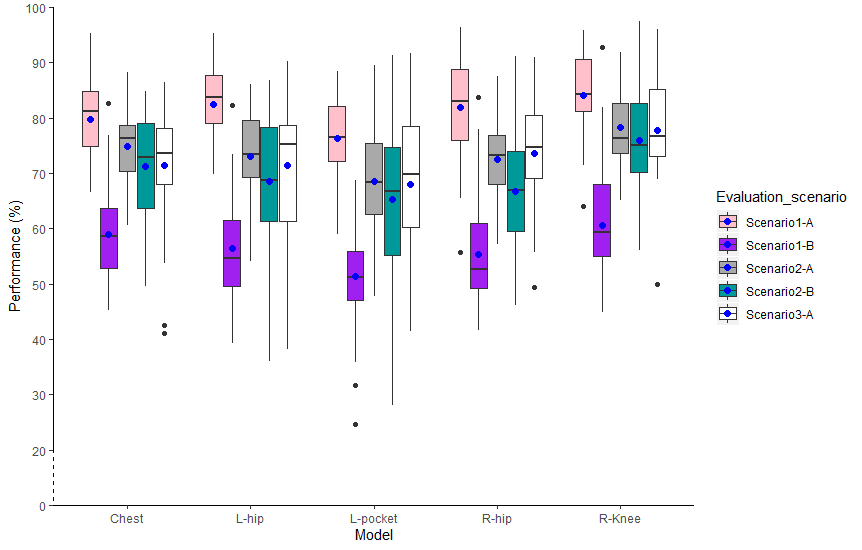
**

**B**

**Figure A4.2.** Distribution of overall accuracy for the individual accelerometer & GPS-based RF classification models. **(A)**: within-old-age models, **(B)**: within-young-age models.

**
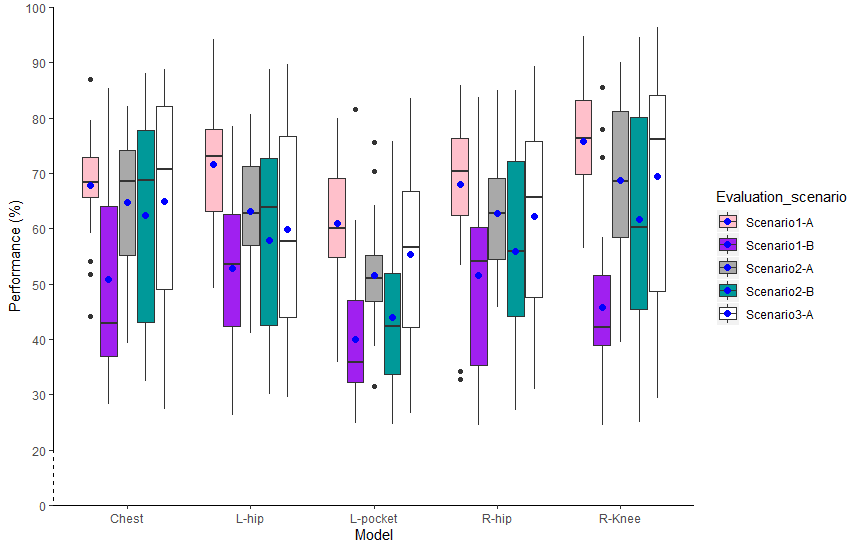
**

**A**

**
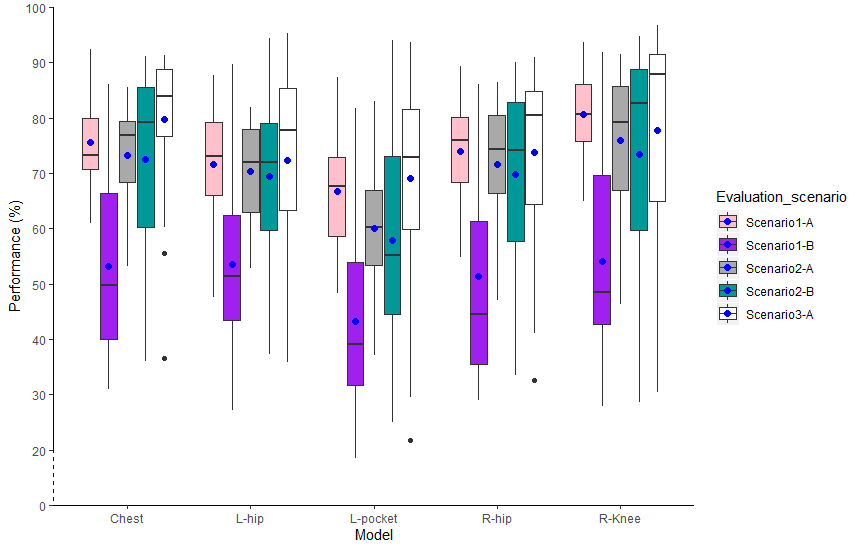
**

**B**

**Figure A4.3.** Distribution of overall accuracy for the individual accelerometer-based RF classification models. **(A)**: Y-trained-btw-age models, **(B)**: Y&O-trained-btw-age models.


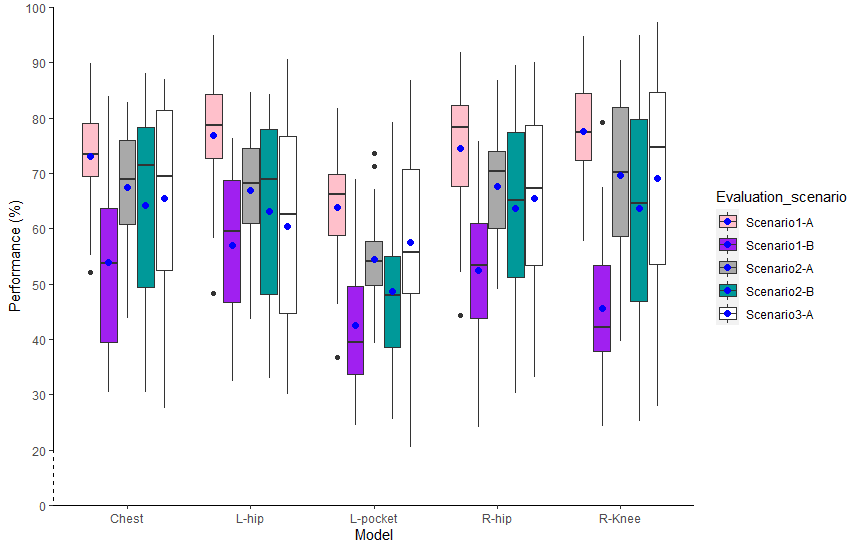


**A**


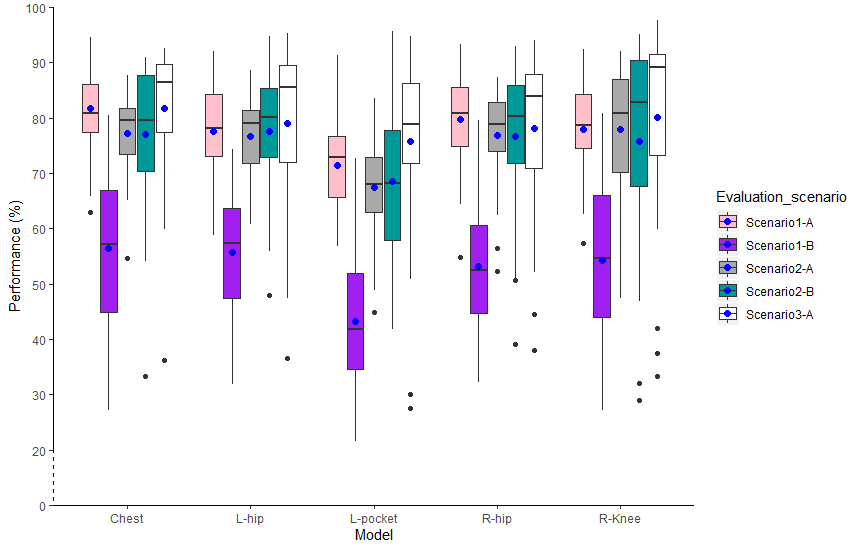


**B**

**Figure A4.4.** Distribution of overall accuracy for the individual accelerometer & GPS-based RF classification models. **(A)**: Y-trained-btw-age models, **(B)**: Y&O-trained-btw-age models.
